# Standing-to-horizontal imaging reveals greater translation than flexion-extension radiographs for assessing lumbar instability in lumbar degenerative spondylolisthesis

**DOI:** 10.1016/j.xnsj.2026.100910

**Published:** 2026-06-01

**Authors:** Alberto Pedrazzini, Marc Schmid, Mazda Farshad, Thorsten Jentzsch

**Affiliations:** Department of Orthopedics, University Spine Center Zurich, Balgrist University Hospital, University of Zurich, Forchstrasse 340, Zurich 8008, Switzerland

**Keywords:** Degenerative spondylolisthesis, Lumbar stenosis, Translation, Instability, Flexion-extension radiographs, CT, MRI, Outcomes

## Abstract

**Background:**

Flexion-extension radiographs are commonly used to assess lumbar instability in degenerative spondylolisthesis, but may underestimate mobility due to limited motion and muscle guarding. This study compared position-dependent translation and instability classification on (1) standing flexion-extension radiographs and (2) standing neutral-to-horizontal computed tomography (CT)/magnetic resonance imaging (MRI), and evaluated associations with postoperative patient-reported outcome measures (PROMS) change.

**Methods:**

In this retrospective single-center cohort study, surgically treated lumbar spinal stenosis patients with degenerative spondylolisthesis were assessed with standing neutral, standing flexion and extension radiographs, and horizontal CT and/or MRI within 6 months preoperatively (n = 227). Outcome measures were translation (millimeters [mm]), instability (Δ translation ≥4 mm), numeric rating scale (NRS) back/leg pain, and Oswestry Disability Index. Translation was measured on each modality. We compared mean translation change for flexion-extension and for standing neutral-to-horizontal CT/MRI. Correlation and agreement (overall agreement; Cohen’s κ) were calculated. PROMS change was analyzed with regression adjusting for surgical technique.

**Results:**

Mean translation change was smaller on flexion-extension radiographs (1.4 ± 2.1 mm) than on standing neutral-to-horizontal comparisons (2.7 ± 2.6 mm for CT and 2.7 ± 2.3 mm for MRI; p < .001). Instability prevalence was 12.8% by flexion-extension and 26.6-30.4% by standing-horizontal comparisons, with limited agreement (overall agreement 62.6%–68.2%, κ ≈ 0). Standing extension translation correlated most strongly with horizontal CT/MRI (r = 0.78 and r = 0.80). PROMs improved at a mean follow-up of 16.1 ± 13.8 months; instability was not independently associated with PROMS change.

**Conclusions:**

Standing neutral-to-horizontal comparisons show larger position-dependent translation and often reclassify instability compared with flexion-extension radiographs. When horizontal CT/MRI is available, routine flexion-extension radiographs may add limited incremental information; standing extension radiographs may be a practical alternative when advanced imaging is unavailable. Imaging-defined instability was not consistently associated with midterm PROMS improvement.

## Introduction

Degenerative lumbar spondylolisthesis frequently coexists with lumbar spinal stenosis and is often evaluated for instability, typically operationalized as abnormal anteroposterior translation between adjacent vertebrae [1]. In clinical practice, instability assessment may influence surgical planning, including decisions between decompression alone and decompression with fusion [2]. Conventional evaluation relies on standing lateral flexion-extension radiographs; however, translation may be underestimated when patient discomfort limits excursion or when increased paraspinal muscle activation in the upright position restricts segmental motion [3,4].

Most patients with lumbar stenosis also undergo horizontal magnetic resonance imaging (MRI), and sometimes computed tomography (CT), as part of routine preoperative workup. Comparing an upright standing neutral radiograph (axial loading) with a horizontal CT/MRI (relative unloading) may reveal position-dependent reduction of slip that is not captured on flexion-extension radiographs. Prior studies suggest that flexion-extension radiographs may underestimate mobility compared with standing-to-horizontal comparisons, but evidence remains limited and heterogeneous, with different thresholds for instability and variable incorporation of patient-reported outcomes [3,[5], [6], [7], [8], [9], [10], [11]].

Translation in millimeters or slip percentage alone provides only a limited description of degenerative spondylolisthesis. Contemporary grading and management frameworks emphasize that sagittal alignment modifiers (eg, segmental lordosis/kyphosis), disc height, and spinopelvic parameters (eg, pelvic incidence and lumbar lordosis) can influence symptoms, progression risk, and surgical strategy beyond slip magnitude alone [12] and are central to the sagittal balance framework in spondylolisthesis [13]. In addition, classification approaches such as the clinical and radiographic degenerative spondylolisthesis (CARDS) system incorporate leg pain, disc space height and sagittal alignment in addition to translation to better stratify degenerative spondylolisthesis subtypes that may differ clinically [14]. Therefore, while the present study focuses on position-dependent translation (a practical and commonly available metric), the results should be interpreted in the context that lordosis and global sagittal alignment were not assessed.

Accordingly, we aimed to (1) quantify position-dependent translation on standing flexion-extension radiographs and on standing neutral-to-horizontal CT/MRI comparisons, (2) evaluate correlation and agreement between modality-dependent definitions of instability, and (3) examine associations between imaging-defined instability and postoperative PROMS changes in surgically treated lumbar stenosis patients with degenerative spondylolisthesis.

## Materials and methods

### Study design and ethics

This retrospective single-center cohort study included surgeries performed from January 2014 through October 2020. The local ethics committee approved the study and waived individual informed consent because of the retrospective design.

### Data source

Clinical and imaging data were extracted from institutional electronic medical records. Imaging was reviewed in the institutional PACS.

### Participants

Patients undergoing surgery for lumbar spinal stenosis with degenerative spondylolisthesis (decompression alone or decompression with fusion) were eligible. Inclusion required all of the following within 6 months before surgery:

1. Lateral standing neutral lumbar radiograph.
2. Standing lateral flexion and standing lateral extension radiographs.
3. At least 1 horizontal advanced imaging study (CT and/or MRI).

**Fig. 1:**
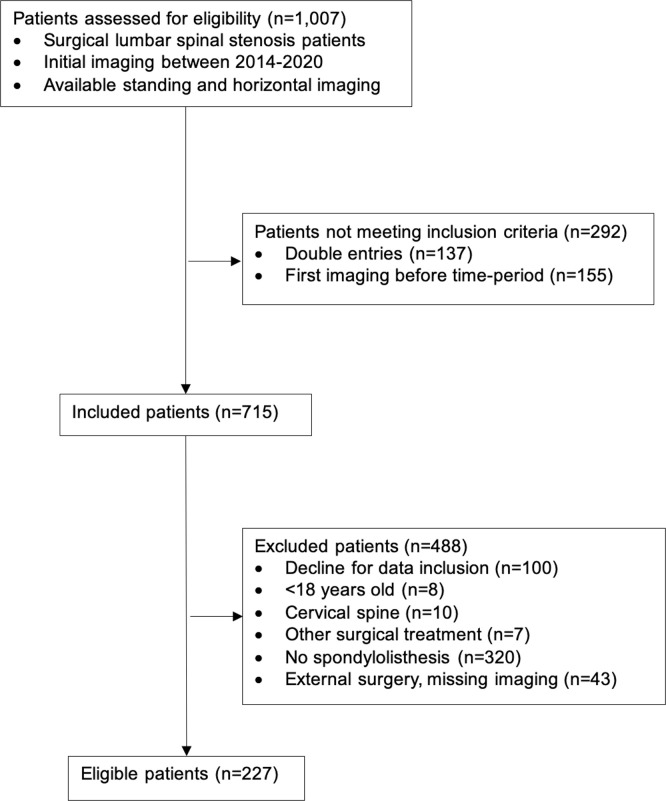
Flowchart of patients (n = 1,007).

Exclusion criteria included age <18 years, cervical pathology, other surgical treatment, absence of spondylolisthesis, external surgery, missing required imaging, or refusal of data use (Fig. 1).

### Imaging and measurement protocol

Anteroposterior translation was measured in millimeters using the posterior vertebral body line (Meyerding method) [15]. Measurements were performed on:

1. Standing neutral lateral radiographs.
2. Standing flexion lateral radiographs.
3. Standing extension lateral radiographs.
4. Horizontal CT (if available).
5. Horizontal MRI (if available).

Index level: Translation was measured at the level of degenerative spondylolisthesis targeted for surgical treatment.

Translation change: For each patient, we calculated the absolute within-patient change in translation (Δtranslation, millimeters [mm]) between:

• Standing flexion versus standing extension radiographs.
• Standing neutral radiograph versus horizontal CT (CT subset).
• Standing neutral radiograph versus horizontal MRI (MRI subset).

**Fig. 2:**
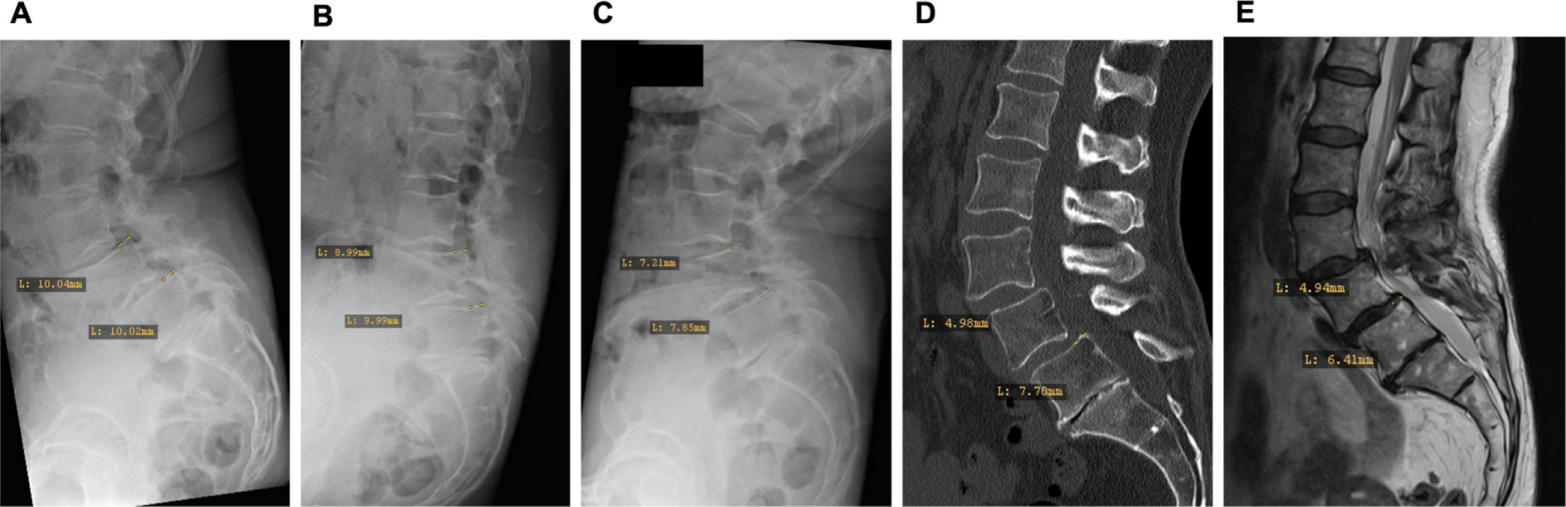
Anteroposterior vertebral body translation at L3/4 and L4/5 in (A) lateral standing neutral radiograph, (B) standing flexion radiograph, (C) standing extension radiograph, (D) horizontal computed tomography (CT), and (E) horizontal magnetic resonance imaging (MRI). There is no instability (translation ≥4 millimeters) in standing flexion/extension radiographs (B vs. C) or in standing neutral radiograph/horizontal CT (A vs. D) and MRI (A vs. E) at L4/5. However, instability can be seen in standing neutral radiograph/horizontal CT (A vs. D) and MRI (A vs. E) at L3/4.

Instability definition: Instability was operationalized as absolute Δtranslation ≥ 4 mm, consistent with prior work using a 4-mm threshold (Fig. 2) [16].

### Surgical technique and decision-making

Surgical strategy (decompression alone vs. decompression with fusion) was determined in a preoperative surgeon consensus conference integrating symptoms, neurological findings, and imaging. Decompression was typically performed through a posterior midline approach with bilateral laminotomies and lateral recess decompression. Fusion procedures generally used posterior pedicle screw instrumentation and a transforaminal lumbar interbody fusion (TLIF) approach with foraminotomy when indicated [17].

### Outcomes

PROMS included:

1. Numeric rating scale (NRS) back pain (0–10).
2. NRS leg pain (0–10).
3. Oswestry Disability Index (ODI) (%).

PROMS were collected preoperatively, approximately 3 months postoperatively, and at final follow-up (≥6 months).

### Statistical analysis

Continuous variables are reported as mean (standard deviation [SD]) and categorical variables as n (%). Analyses were performed within available subsets (CT n = 131; MRI n = 214; both CT+MRI n = 118). Absolute translation change between imaging positions was assessed using paired tests (paired t-test or Wilcoxon signed-rank test as appropriate). Correlations between continuous translation measures were evaluated using Pearson correlation (r). Agreement for dichotomized instability (Δtranslation ≥4 mm) between flexion-extension and horizontal imaging definitions was quantified using overall percent agreement and Cohen’s κ with 95% confidence intervals. Associations between instability and PROMS change were evaluated using linear regression adjusting for surgical technique (decompression vs. decompression plus fusion). A 2-sided p-value <.05 was considered statistically significant. Analyses were performed in Stata.

## Results

### Cohort characteristics

A total of 227 patients met inclusion criteria. Mean age was 65.2 ± 13.9 years; 64.8% were female. Mean BMI was 27.2 ± 4.9 kilograms (kg)/meter (m)2. The most commonly affected level was L4/5 (50.7%), followed by L5/S1 (32.6%). About 17.6% underwent decompression alone and 82.4% underwent decompression with fusion (Table 1).

**Table 1:** Baseline patient data (n = 227).

Variables	Mean	(SD)
Age (y)	65.2	(13.9)
Female gender (n [%])	147	(64.8)
BMI (kg/m^2^) (n = 224)	27.2	(4.9)
Segment (n [%])		
L1/2	3	(1.3)
L2/3	5	(2.2)
L3/4	30	(13.2)
L4/5	115	(50.7)
L5/S1	74	(32.6)
Surgical technique (n [%])		
Decompression only	45	(17.6)
Decompression plus fusion	187	(82.4)

SD, standard deviation; n, number; BMI, body mass index; kg, kilograms; m, meters.

### Imaging measurements

Mean translation was 9.2 ± 4.0 mm on standing neutral radiographs, 10.2 ± 3.8 mm on standing flexion, and 8.8 ± 4.1 mm on standing extension. Horizontal translation was 7.3 ± 3.3 mm on CT (n = 131) and 6.6 ± 3.1 mm on MRI (n = 214).

### Translation change across positions

Mean absolute translation change on standing flexion-extension radiographs was 1.4 ± 2.1 mm. Translation change between standing neutral radiographs and horizontal imaging was larger: 2.7 ± 2.6 mm for standing neutral versus horizontal CT and 2.7 ± 2.3 mm for standing neutral versus horizontal MRI (Table 2).

**Table 2:** Imaging data (n = 227).

Variables	Mean	(SD)	p-value*
Translation (anteroposterior)			
Standing radiographs			
Neutral	9.2	(4.0)	
Flexion	10.2	(3.8)	
Extension	8.8	(4.1)	
Horizontal imaging			
CT (n = 131)	7.3	(3.3)	
MRI (n = 214)	6.6	(3.1)	
Differences in imaging			
Standing flexion radiograph versus standing extension	1.4	(2.1)	
Standing neutral radiograph versus horizontal CT (n = 131)	2.7	(2.6)	
Standing neutral radiograph versus horizontal MRI (n = 214)	2.7	(2.3)	
Instability (translation ≥4 mm) (n [%])			
Standing flexion versus standing extension	29	(12.8)	
Standing neutral radiograph versus horizontal CT (n = 131)	37	(28.2)	
Standing neutral radiograph versus horizontal MRI (n = 214)	57	(26.6)	
Standing neutral radiograph versus horizontal CT or horizontal MRI	69	(30.4)	
Pearson correlation (r) between imaging modalities			
Standing neutral radiograph versus horizontal CT (n = 131)	0.22		.012
Standing neutral radiograph versus horizontal MRI (n = 214)	0.12		.07
Standing flexion radiograph versus horizontal CT (n = 131)	0.59		<.001
Standing extension radiograph versus horizontal CT (n = 131)	0.78		<.001
Standing flexion radiograph versus horizontal MRI (n = 214)	0.67		<.001
Standing extension radiograph versus horizontal MRI (n = 214)	0.80		<.001
Horizontal CT versus horizontal MRI (n = 118)	0.80		<.001
Instability (translation ≥4 mm) and surgical technique (n [%])			
Instability based on standing flexion radiograph versus standing extension radiograph			.32
Decompression only	7	(24.1)	
Decompression plus fusion	22	(75.9)	
Instability based on standing neutral radiograph and horizontal CT (n = 131)			.62
Decompression only	5	(13.5)	
Decompression plus fusion	32	(86.5)	
Instability based on standing neutral radiograph and horizontal MRI (n = 214)			.23
Decompression only	6	(10.5)	
Decompression plus fusion	51	(89.5)	
Instability based on standing neutral radiograph and horizontal CT or horizontal MRI			.66
Decompression only	11	(15.9)	
Decompression plus fusion	58	(84.1)	

SD, standard deviation; n, number; CT, computed tomography; MRI, magnetic resonance imaging; mm, millimeters.

⁎ Pearson correlation (r) and chi-squared test.

### Instability prevalence by imaging comparison

Instability prevalence (Δtranslation ≥ 4 mm) varied by the imaging comparison used (Table 2):

• About 12.8% (29/227) for standing flexion versus standing extension.
• About 28.2% (37/131) for standing neutral versus horizontal CT.
• About 26.6% (57/214) for standing neutral versus horizontal MRI.
• About 30.4% (69/227) when defining instability using either available horizontal modality.

### Correlation between modalities

Standing extension translation correlated most strongly with horizontal CT and MRI (r = 0.78 and r = 0.80, respectively; both p < .001). Standing flexion correlated moderately with horizontal CT and MRI (r = 0.59 and r = 0.67; both p < .001). Standing neutral showed weak correlation with horizontal CT (r = 0.22; p = .012) and horizontal MRI (r = 0.12; p = .07). In patients with both horizontal modalities (n = 118), CT and MRI translation correlated strongly (r = 0.80; p < .001) (Table 2).

### Agreement in instability classification

Overall agreement between flexion-extension-defined instability and horizontal imaging-defined instability was 62.6% for CT, 68.2% for MRI, and 63.0% when using CT or MRI. Cohen’s κ values were near zero (κ = −0.07 for CT, κ = 0.01 for MRI, κ = −0.05 for CT or MRI; 95% confidence intervals crossing zero), indicating minimal agreement beyond chance (Table 3).

**Table 3:** Agreement of instability (≥4 millimeter translation) on imaging data (n = 227).

Variable	Agreement (%)	κ (95% CI)
Standing flexion/extension radiograph versus horizontal CT (n = 131)	62.6	−0.07 (95% CI −0.23 to 0.09, p = .81)
Standing flexion/extension radiograph versus horizontal MRI (n = 214)	68.2	0.01 (95% CI −0.11 to 0.13, p = .43)
Standing flexion/extension radiograph versus horizontal CT or horizontal MRI	63.0	−0.05 (−0.16 to 0.07, p = .78)

%, percent; κ, Cohen’s kappa; CI, confidence interval; CT, computed tomography; MRI, magnetic resonance imaging.

Standing flexion versus standing extension radiographs were considered gold-standard.

### Instability and surgical technique

Among patients meeting the instability definition, most underwent decompression with fusion across all comparisons. The distribution of surgical technique among unstable patients did not differ significantly across the imaging definitions used (Table 2).

## PROMS

PROMS improved after surgery at mean follow-up 16.1 ± 13.8 months. Back pain decreased from 6.6 ± 2.1 preoperatively to 3.3 ± 2.3 at ∼3 months and 4.0 ± 2.6 at ≥6 months. Leg pain decreased from 6.1 ± 2.7 preoperatively to 2.5 ± 2.6 at ∼3 months and 3.2 ± 3.0 at ≥6 months. ODI decreased from 19.6% ± 8.3% preoperatively to 14.0% ± 10.0% at ∼3 months and 10.6% ± 7.9% at ≥6 months (Table 4).

**Table 4:** Clinical data (n = 168).

Variables	Mean	(SD)	P-value*
PROMS†			
Back pain (points‡)			
Preoperative (n = 146)	6.6	(2.1)	
Postoperative (∼3 mo) (n = 64)	3.3	(2.3)	
Postoperative (>6 mo) (n = 82)	4.0	(2.6)	
Leg pain (points‡)			
Preoperative (n = 147)	6.1	(2.7)	
Postoperative (∼3 mo) (n = 64)	2.5	(2.6)	
Postoperative (>6 mo) (n = 82)	3.2	(3.0)	
ODI (%)			
Preoperative	19.6	(8.3)	
Postoperative (∼3 mo) (n = 58)	14.0	(10.0)	
Postoperative (>6 mo) (n = 73)	10.6	(7.9)	
Differences			
Back pain (points)			
Preoperative versus postoperative (∼3 mo) (n = 45)	−2.6	(2.8)	<.001
Preoperative versus postoperative (>6 mo) (n = 64)	−2.3	(3.1)	<.001
Leg pain (points)			
Preoperative versus postoperative (∼3 mo) (n = 45)	−3.8	(3.4)	<.001
Preoperative versus postoperative (>6 mo) (n = 64)	−3.0	(3.8)	<.001
ODI (%)			
Preoperative versus postoperative (∼3 mo) (n = 52)	−4.6	(9.3)	<.001
Preoperative versus postoperative (>6 mo) (n = 62)	−7	(9.8)	<.001

PROMS, patient-reported outcome measures; SD, standard deviation; n, number; ODI, Oswestry Disability Index.

⁎ Paired t-test.

† Mean follow up of 16.1 (standard deviation 13.8) months.

‡ Numeric rating scale from 0 (none) to 10 (worst).

### PROMS change by instability

Across imaging definitions, instability was not consistently associated with pre- to postoperative changes in NRS back pain, NRS leg pain, or ODI. Any unadjusted differences in ODI at ≥6 months attenuated after adjustment for surgical technique and were not consistently statistically significant (Table 5).

**Table 5:** Clinical and imaging data for instability (n = 227).

Variables	Instability					
	No		Yes			
	Mean	(SD)	Mean	(SD)	p-value*	Coefficient (95% CI, p-value)†
PROMS and flexion/extension						
Back pain (points‡)						
Pre- versus postoperative (∼3 mo) (n = 45)	−2.7	(2.8)	−2.5	(2.4)	.89	0.2 (−2.8 to 3.2, .88)
Pre- versus postoperative (>6 mo) (n = 64)	−2.7	(2.8)	−1.1	(4.3)	.20	1.4 (−1.1 to 3.8, .26)
Leg pain (points‡)						
Pre- versus postoperative (∼3 mo) (n = 45)	−3.9	(3.5)	−2.8	(2.2)	.54	1.6 (−2.0 to 5.2, .36)
Pre- versus postoperative (>6 mo) (n = 64)	−3.1	(4.0)	−4.6	(3.2)	.36	−1.6 (−4.8 to 1.6, .31)
ODI (%)						
Pre- versus postoperative (∼3 mo) (n = 52)	−4.5	(9.5)	−3	(6.4)	.76	4.9 (−8.6 to 11.1, .80)
Pre- versus postoperative (>6 mo) (n = 62)	−8.0	(9.6)	5.0	(8.5)	.02	10.8 (−1.0 to 22.6, .07)
PROMS and standing neutral radiograph/CT						
Back pain (points‡)						
Pre- versus postoperative (∼3 mo) (n = 24)	−2.8	(3.0)	−1.8	(2.8)	.51	1.1 (−1.8 to 4.1, .44)
Pre- versus postoperative (>6 mo) (n = 37)	−2.7	(3.0)	−2.2	(3.5)	.65	0.6 (−1.8 to 3.0, .61)
Leg pain (points‡)						
Pre- versus postoperative (∼3 mo) (n = 24)	−3.9	(3.9)	−3.5	3.6	.81	0.6 (−3.3 to 3.3, .77)
Pre- versus postoperative (>6 mo) (n = 37)	−3.1	(4.5)	−3.2	(3.7)	.97	−0.1 (−3.4 to 3.2, .97)
ODI (%)						
Pre- versus postoperative (∼3 mo) (n = 32)	−3.8	(9.9)	−2.0	(10.8)	.65	1.6 (−6.6 to 9.8, .69)
Pre- versus postoperative (>6 mo) (n = 41)	−9.7	(9.6)	−2.9	(10.3)	.06	7.1 (−0.02 to 14.1, .051)
PROMS and standing neutral radiograph/MRI						
Back pain (points‡)						
Pre- versus postoperative (∼3 mo) (n = 44)	−3.2	(2.8)	−1.6	(32.3)	.08	1.6 (−0.2 to 3.4, .09)
Pre- versus postoperative (>6 mo) (n = 63)	−2.7	(2.7)	−21.9	(3.8)	.37	0.7 (−0.9 to 2.4, .38)
Leg pain (points‡)						
Pre- versus postoperative (∼3 mo) (n = 44)	−4.1	(3.4)	−3.5	(2.8)	.59	0.6 (−1.6 to 2.8, .60)
Pre- versus postoperative (>6 mo) (n = 63)	−3.6	(4.0)	−2.7	(3.9)	.41	0.9 (−1.3 to 3.1, .42)
ODI (%)						
Pre- versus postoperative (∼3 mo) (n = 51)	−4.8	(9.1)	−3.4	(10.0)	.60	1.3 (−4.2 to 6.8, .64)
Pre- versus postoperative (>6 mo) (n = 61)	−5.2	(11.1)	−5.2	(11.1)	.32	1.9 (−3.8 to 7.6, .50)
PROMsS and standing neutral radiograph/CT or MRI						
Back pain (points‡)						
Pre- versus postoperative (∼3 mo) (n = 45)	−3.1	(2.8)	−1.8	(2.3)	.15	1.3 (−0.5 to 3.1, .16)
Pre- versus postoperative (>6 mo) (n = 64)	−2.8	(2.8)	−2.0	(3.5)	.27	0.8 (−0.8 to 2.4, .34)
Leg pain (points‡)						
Pre- versus postoperative (∼3 mo) (n = 45)	−3.8	(3.7)	−3.5	(2.7)	.79	0.3 (−2.0 to 2.5, .82)
Pre- versus postoperative (>6 mo) (n = 64)	−3.7	(3.9)	−2.5	(4.0)	.28	1.1 (−1.0 to 3.1, p = 0.32)
ODI (%)						
Pre- versus postoperative (∼3 mo) (n = 52)	−5.5	(8.7)	−2.7	(10.2)	.30	2.6 (−2.7 to 8.0, p = 0.33)
Pre- versus postoperative (>6 mo) (n = 62)	−9.4	(9.0)	−3.8	(10.9)	.036	4.9 (−2.7 to 10.1, p = 0.063)

SD, standard deviation; CI, confidence interval; PROMS, patient-reported outcome measures; ODI, Oswestry Disability Index; CT, computed tomography; MRI, magnetic resonance imaging.

⁎ Unpaired t-test.

† Linear regression model adjusting for the surgical technique (ie, decompression versus decompression plus fusion).

‡ Numeric rating scale from 0 (none) to 10 (worst).

## Discussion

This retrospective single-center cohort study evaluated position-dependent measures of translation in 227 surgically treated patients with lumbar spinal stenosis and degenerative spondylolisthesis. The principal findings were: (1) mean absolute translation change was smaller on standing flexion-extension radiographs (1.4 ± 2.1 mm) than on standing neutral-to-horizontal comparisons (2.7 ± 2.6 mm for CT; 2.7 ± 2.3 mm for MRI); (2) instability prevalence (Δtranslation ≥4 mm) differed substantially depending on which imaging comparison was used (12.8% on flexion-extension vs. 26.6%–30.4% on standing-to-horizontal comparisons); (3) agreement between flexion-extension-defined instability and horizontal-imaging-defined instability was limited (κ ≈ 0 despite 62.6%–68.2% agreement); and (4) imaging-defined instability was not consistently associated with midterm PROMS improvement.

### Interpretation in the context of prior work

Our findings add to a small but growing body of literature questioning whether standing flexion-extension radiographs fully capture translation behavior in degenerative spondylolisthesis. Lee et al. reported that flexion-extension radiographs may underestimate mobility compared with flexion-to-horizontal comparisons, emphasizing the potential role of standing-to-horizontal changes in revealing positional reduction [3]. Chan et al. similarly contrasted flexion-extension radiographs with standing neutral radiographs and horizontal MRI and highlighted modality-dependent differences in instability classification [6]. Studies examining standing versus horizontal slippage have also suggested that the mechanical state (loaded vs. unloaded) affects measured translation [7]. Collectively, these reports align with the concept that instability classification is sensitive to posture, patient effort, and muscle tone, and that flexion-extension views may not reflect the maximal position-dependent change that can be observed when comparing loaded standing to unloaded horizontal imaging.

At the same time, posture-dependent evaluation of translation extends beyond standard flexion-extension. Investigations of sitting or other postures suggest that alternative functional positions may better reveal mobility in selected patients [[8], [9], [10], [11]]. These studies support a broader interpretation that there may not be a single universally optimal posture for identifying clinically relevant motion; rather, different modalities probe different mechanical states and may yield different but potentially complementary information.

### Why standing-horizontal may show larger change than flexion-extension?

A clinically plausible explanation for larger standing-to-horizontal translation change is that horizontal imaging reflects partial reduction of slip due to unloading and reduced paraspinal activation, whereas flexion-extension radiographs depend on patient tolerance, effort, and the ability to reach end-range postures. Pain-limited excursion and guarding can reduce measurable translation on flexion-extension views, particularly in symptomatic stenosis patients [3,5]. This interpretation is consistent with the concept of hidden or posture-dependent spondylolisthesis described in prior work, in which translation may differ between upright and recumbent conditions [5].

### Correlation findings

Standing extension translation showed the strongest correlation with horizontal CT and MRI (r = 0.78 and 0.80), suggesting that extension radiographs may approximate the alignment observed in the unloaded horizontal position. A pragmatic implication is that when advanced imaging is not yet available at an initial evaluation, an extension lateral view may provide a closer proxy for horizontal translation than neutral or flexion views. However, correlation does not imply interchangeability, and standardized acquisition would be required before recommending extension views as a substitute.

### Agreement

Although overall agreement between flexion-extension-defined and standing-to-horizontal-defined instability was moderate (62.6%–68.2%), κ values were near zero. This apparent contradiction can occur when the prevalence of positive classifications differs meaningfully between the 2 methods (here, 12.8% vs. 26.6%–30.4%). In such circumstances, κ may be low despite nontrivial raw agreement because agreement is adjusted for chance and is sensitive to marginal totals (prevalence). Practically, the low κ supports the core point: the 2 approaches frequently classify the same patients differently, and thus should not be assumed interchangeable.

### Clinical implications for imaging strategy

From a workflow standpoint, most patients with lumbar stenosis already undergo standing neutral radiographs and horizontal MRI to characterize stenosis, neural compression, and foraminal pathology. In that common scenario, the incremental value of routinely adding flexion-extension radiographs solely to classify translation-based instability may be limited. A standing neutral radiograph paired with routine horizontal CT/MRI may capture a larger position-dependent translation signal than flexion-extension radiographs and may identify patients whose slip meaningfully reduces when unloaded. This observation does not establish diagnostic superiority, because no definitive reference standard exists for true instability in degenerative spondylolisthesis [1]. Rather, it suggests that standing-to-horizontal comparisons can provide substantial information without requiring additional dynamic radiographic acquisition in many patients.

At the same time, imaging is only 1 input into surgical planning. Current guidelines and evidence underscore that symptoms, functional limitation, stenosis severity, back pain phenotype, and foraminal compromise contribute to the decompression versus fusion decision [2]. The high proportion of fusion procedures in this cohort despite a lower proportion meeting the flexion-extension instability threshold supports the reality that translation cutoffs alone may not be the dominant driver in surgical strategy selection.

### Slip magnitude and sagittal alignment considerations

Simple slip magnitude (or slip percentage) is only 1 radiographic descriptor of degenerative spondylolisthesis. Modern grading perspectives emphasize that segmental alignment (eg, segmental lordosis/kyphosis modifiers), disc height, and spinopelvic context can meaningfully affect symptom profiles, progression risk, and operative planning beyond translation alone [12]. Similarly, classification systems such as CARDS incorporate leg pain, disc space height and sagittal alignment in addition to translation to differentiate phenotypes that may have distinct clinical behavior [14]. Moreover, cross-sectional imaging may provide additional features linked to instability beyond translation (eg, facet joint effusion on MRI), which we did not incorporate into our definition [18]. In that context, our findings should be interpreted as evaluating modality-dependent translation and translation-based instability definitions rather than providing a comprehensive characterization of degenerative spondylolisthesis severity or sagittal balance.

### PROMS and why instability may not predict outcome well

PROMS improved substantially after surgery, but imaging-defined instability was not consistently associated with PROMS change. This finding is consistent with the multifactorial determinants of postoperative improvement in lumbar stenosis with degenerative spondylolisthesis. Randomized and comparative data (including work using a 4-mm instability framing in surgical trials) show that outcomes are influenced by patient selection, symptom profile, stenosis severity, and procedure type, rather than translation threshold alone [16]. Meta-analytic evidence comparing decompression versus fusion also indicates that differences in outcomes can be nuanced and patient-dependent [4]. Thus, a lack of consistent association between a single translation-based instability definition and PROMS improvement is plausible, particularly when patients undergo differing procedures for varied clinical indications.

### Limitations

This study has limitations inherent to its retrospective design, including possible confounding by indication and incomplete PROMS data in some patients. The imaging window (within 6 months preoperatively) introduces potential variability due to symptom fluctuation or progression. Measurements were performed by a single trained rater; while prior studies report acceptable reliability for similar translation measures, a formal inter-/intrarater reliability analysis would strengthen reproducibility [6]. Furthermore, instability was operationalized using a 4-mm cutoff based on prior literature; however, thresholds vary across studies (eg, 3 mm or percentage-based definitions) [16]. Finally, no universal reference standard exists for radiographic instability in degenerative spondylolisthesis; therefore, our findings should be interpreted as describing differences, correlations, and agreement between modality-dependent definitions, not as establishing diagnostic accuracy [1].

An additional limitation is the absence of segmental and global sagittal alignment assessment. We did not quantify segmental lordosis at the index level, overall lumbar lordosis, pelvic incidence, or mismatch parameters (eg, pelvic incidence minus lumbar lordosis), which are increasingly recognized as important contextual factors in degenerative spondylolisthesis grading and management [[12], [13], [14]]. Because lordosis and spinopelvic alignment may interact with translation/reducibility and may influence symptoms and outcomes, our translation-focused analyses cannot determine whether the observed modality-dependent differences are modified by sagittal alignment.

### Future directions

Prospective studies with standardized acquisition (including controlled flexion/extension effort, sitting radiographs where appropriate, and consistent CT/MRI measurement planes) and multi-reader reliability would clarify how best to integrate posture-dependent imaging into decision-making. Future work should also test whether certain clinical phenotypes (eg, predominant mechanical back pain, marked reducibility, or severe foraminal stenosis) derive greater benefit from one imaging strategy over another, and whether modality-dependent translation measures predict clinically meaningful endpoints (eg, minimally clinically important difference (MCID) responder status for ODI or pain). Furthermore, future prospective work could integrate translation, slip percentage, and segmental/global lordosis measures in a unified model to clarify which combinations best predict clinically meaningful outcomes and inform procedure selection [[12], [13], [14]].

## Conclusion

Standing neutral-to-horizontal comparisons show larger position-dependent translation and often reclassify instability compared with flexion-extension radiographs. When horizontal CT/MRI is available, routine flexion-extension radiographs may add limited incremental information; standing extension radiographs may be a practical alternative when advanced imaging is un-available. Imaging-defined instability was not consistently associated with midterm PROMS improvement.

## Declaration of generative AI AND AI-assisted technologies in the manuscript preparation process

During the preparation of this work the authors used ChatGPT; OpenAI in order to support manuscript drafting and language editing (eg, improving clarity, grammar, and structure). After using this tool/service, the authors reviewed and edited the content as needed and takes full responsibility for the content of the published article.

## Ethical approval

This study was approved by the local ethics committee (KEK 2021-01529), who waived individual consent due to the retrospective study.

## Funding

The AO Spine North America (AO SNA) and the Research Committee awarded the grant application “New Definition of Instability and Indication for Fusion Surgery: Standing Versus Supine Imaging” with funding ($10,000.00) as one of their Young Investigator Research Grant Awards in 2021.

## Author contributions

AP: acquisition of data, analysis and interpretation of data, drafting the manuscript; MS: acquisition of data; MF: conception and design, drafting the manuscript; TJ: idea, conception and design, analysis and interpretation of data, drafting the manuscript; all: revision of the manuscript, final approval of the version to be published.

## Declaration of competing interests

The authors declare that they have no known competing financial interests or personal relationships that could have appeared to influence the work reported in this paper.
